# Loss of microbial signals reprograms endocrine microenvironments and consistently reduces RESISTIN expression in the adrenal and thyroid cells of germ-free pigs

**DOI:** 10.1016/j.gendis.2026.102078

**Published:** 2026-02-13

**Authors:** Jingjing Tian, Teng Wang, Ting Zhou, Yu Pan, Haitao Shang, Song Li, Shengjun Yang, Li Li, Shuai Tan, Gaohui Zhu

**Affiliations:** Department of Endocrinology Children's Hospital of Chongqing Medical University, National Clinical Research Center for Children and Adolescents' Health and Diseases, Ministry of Education Key Laboratory of Child Development and Disorders, International Science and Technology Cooperation Base of Child Development and Critical Disorders, Chongqing Key Laboratory of Child Rare Diseases in Infection and Immunity, Chongqing 400015, China; Yu-Yue Pathology Scientific Research Center, Jinfeng Laboratory, Chongqing 401329, China; Department of Bioinformatics, College of Artificial Intelligence Medicine, Chongqing Medical University, Chongqing 400016, China; Department of Endocrinology Children's Hospital of Chongqing Medical University, National Clinical Research Center for Children and Adolescents' Health and Diseases, Ministry of Education Key Laboratory of Child Development and Disorders, International Science and Technology Cooperation Base of Child Development and Critical Disorders, Chongqing Key Laboratory of Child Rare Diseases in Infection and Immunity, Chongqing 400015, China; Yu-Yue Pathology Scientific Research Center, Jinfeng Laboratory, Chongqing 401329, China; Precision Medicine Institute, The First Affiliated Hospital, Sun Yat-sen University, Guangzhou, Guangdong 510080, China; Department of Neurosurgery, Xinqiao Hospital, Army Medical University, Chongqing 400037, China; Department of Endocrinology Children's Hospital of Chongqing Medical University, National Clinical Research Center for Children and Adolescents' Health and Diseases, Ministry of Education Key Laboratory of Child Development and Disorders, International Science and Technology Cooperation Base of Child Development and Critical Disorders, Chongqing Key Laboratory of Child Rare Diseases in Infection and Immunity, Chongqing 400015, China

In studies of germ-free organisms, significant differences in adrenal and thyroid gland functions have been observed, particularly in murine models.^1^^,^^2^ Mice, being nocturnal animals, exhibit distinct biological rhythms that differ from humans, which may lead to confounding factors when studying the effects of microbiota absence on endocrine functions.^3^^,^^4^ In contrast, pigs, with their more comparable circadian rhythm to humans, represent a more suitable model for investigating the impact of a germ-free environment on endocrine organ function. To explore this further, we selected one-month-old germ-free (GF) and specific-pathogen-free (SPF) pigs for a comprehensive study. Recent studies show that the microbiota helps shape the development of mammalian endocrine glands by regulating early-life hormone production, metabolic signals, and immune pathways.^5^ We focused on the adrenal and thyroid glands, performing single-cell RNA sequencing and integrated analyses to investigate the cellular and molecular differences between these two groups.

Through quality control analysis (Fig. S1A–S1H) and cell type annotation of single-cell transcriptomic data (Fig. S2A–S2C), we ensured the accuracy and reliability of the identified cell populations and gene expression profiles. The adrenal and thyroid single-cell atlases are shown in Figure 1A and B, respectively. In the adrenal glands, cortical cells represent the largest population in GF pigs (50.8%), followed by fibroblasts (19.5%) and endothelial cells (11.0%) (Fig. 1C). In contrast, SPF pigs show a marked reduction in cortical cells (18.8%) and a significant increase in endothelial cells (32.0%). Notably, SPF pigs also have higher proportions of T cells (16.2%) and neutrophils (8.1%) compared to GF pigs. In the thyroid gland, the predominant population in GF pigs consists of follicular epithelial cells (46.5%) and endothelial cells (22.9%) (Fig. 1C). SPF pigs exhibit a marked increase in follicular epithelial cells (68.6%), with a decrease in T cells (3.8%), endothelial cells (4.3%), and other immune cells.

**Figure 1:**
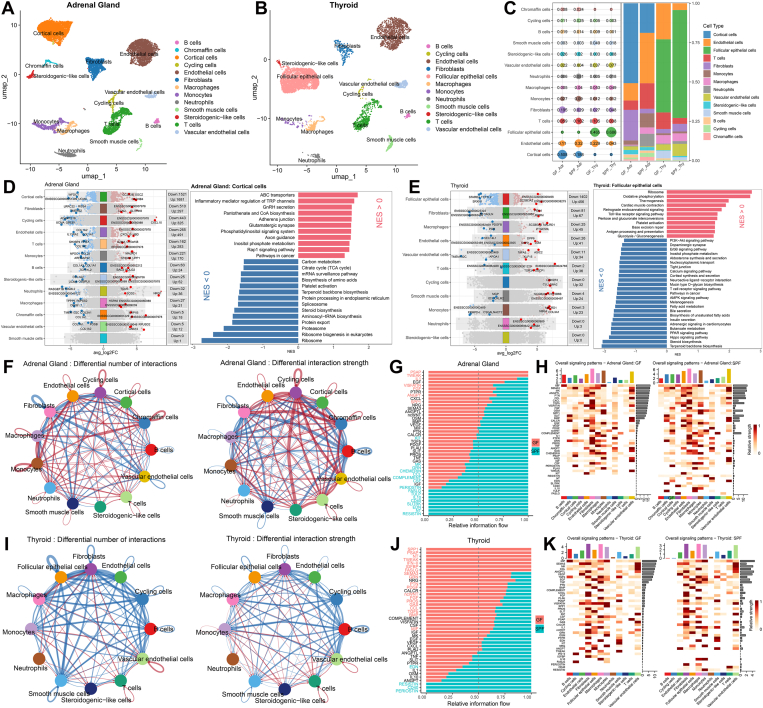
Single-cell RNA sequencing and integrative analysis of adrenal gland and thyroid gland in one-week-old GF and SPF pigs. **(A)** Adrenal single-cell atlas from specific-pathogen-free (SPF) pigs and germ-free (GF) pigs. **(B)** Thyroid single-cell atlas from SPF pigs and GF pigs. **(C)** Proportions of different cell types across SPF and GF pig samples. The size of the dots and the length of the bars represent the relative proportions of each cell type within the sample. **(D)** Differential analysis of cell types in the adrenal glands between GF and SPF pigs, showing the top 3 most up-regulated and down-regulated genes. Bar charts display the KEGG pathways significantly enriched in adrenal cortical cells based on Gene Set Enrichment Analysis (GSEA, *p* < 0.05). The red color represents the enrichment results for up-regulated genes in the comparison of GF *vs.* SPF pigs, while the blue color represents the enrichment results for down-regulated genes in the same GF *vs.* SPF pig comparison. **(E)** Differential analysis of cell types in the thyroid glands between GF and SPF pigs, showing the top 3 most up-regulated and down-regulated genes. Bar charts display the KEGG pathways significantly enriched in thyroid follicular epithelial cells based on GSEA (*p* < 0.05). The red color represents the enrichment results for up-regulated genes in the comparison of GF *vs.* SPF pigs, while the blue color represents the enrichment results for down-regulated genes in the same GF *vs.* SPF pig comparison. **(F)** Differences in the number and strength of intercellular communication in the adrenal glands between the GF and SPF pigs. Red indicates an increase or enhancement in GF pigs, and blue indicates a decrease or weakening in GF pigs. **(G)** Bar chart showing the differential enrichment of signaling pathways between the GF and SPF adrenal groups. Red on the vertical axis indicates a significant increase in the GF pigs, blue indicates a significant increase in the SPF pigs, and black indicates no significant difference. **(H)** Heatmap of intercellular signaling pathways in the adrenal glands of SPF and GF pigs. The vertical axis represents signaling pathways, and the horizontal axis represents cell subtypes. The intensity of color represents the communication strength (darker colors indicate higher intensity). Bar charts above and to the right indicate the cumulative communication strength along the horizontal and vertical axes, respectively. **(I)** Differences in the number and strength of intercellular communication in the thyroid glands between GF and SPF pigs. Red indicates an increase or enhancement, and blue indicates a decrease or weakening. **(J)** Bar chart showing the differential enrichment of signaling pathways between the GF and SPF thyroid groups. Red on the vertical axis indicates a significant increase in the GF group, blue indicates a significant increase in the SPF group, and black indicates no significant difference. **(K)** Heatmap of intercellular signaling pathways in the thyroid glands of SPF and GF pigs. The vertical axis represents signaling pathways, and the horizontal axis represents cell subtypes. The intensity of color represents the communication strength (darker colors indicate higher intensity). Bar charts above and to the right indicate the cumulative communication strength along the horizontal and vertical axes, respectively.

Single-cell transcriptome analysis revealed distinct gene expression alterations in various cell types (Fig. 1D). In B cells, extracellular matrix-related genes like COL1A1 and COL1A2 were down-regulated. In chromaffin cells, LRP1B was up-regulated, but collagen-related genes (COL1A1, COL3A1) were also down-regulated. These findings suggest that the reduced expression of collagen and other ECM components in GF animals may weaken matrix stability and cell–matrix adhesion, potentially disrupting the structural support and functional microenvironment required for optimal steroidogenesis and thyroid hormone production. Gene Set Enrichment Analysis (GSEA) revealed significant differences in signaling pathways between GF and SPF pigs. In adrenal cortical cells, SPF pigs displayed activation of pathways associated with cell signaling, such as ABC transporters, inflammatory mediator regulation of TRP channels, and phosphatidylinositol signaling (Fig. 1D). Conversely, pathways related to protein synthesis and steroid metabolism, including steroid biosynthesis and the TCA cycle, were notably down-regulated in SPF pigs. These changes suggest a shift in adrenal cell functions from steroidogenesis to enhanced signaling and structural maintenance. In thyroid follicular epithelial cells, GSEA highlighted the up-regulation of protein synthesis and energy metabolism pathways, including oxidative phosphorylation and glycolysis (Fig. 1E). Conversely, lipid and steroid metabolism pathways, including PPAR signaling and bile secretion, were suppressed, suggesting a shift toward weakened immune response and energy production in GF pigs.

Analysis of cell-to-cell communication revealed an increase in communication events among macrophages, monocytes, neutrophils, and T cells in the SPF adrenal microenvironment (Fig. 1E). In contrast, fibroblasts, vascular endothelial cells, and smooth muscle cells showed decreased communication (Fig. 1F and G). Notably, the strongest communication signals in the adrenal glands of GF pigs were mediated by MIF and directed mainly at monocytes, macrophages, and B cells (Fig. 1H). In SPF pigs, MIF signaling shifted towards monocytes, macrophages, and cycling cells, with weaker signaling in B cells. The communication signals for the thyroid show that the most significant change is the decreased communication strength between B cells and other cell types. Additionally, a noticeable decrease in signaling between cycling cells and other cell types was observed (Fig. 1I–K). Notably, the consistently higher RESISTIN expression observed in SPF pigs in both adrenal and thyroid tissues (Fig. 1G–J) suggests microbiota-driven enhancement of immune and metabolic activity, whereas the uniformly reduced RESISTIN levels in GF pigs likely reflect diminished basal immune tone and metabolic reprogramming in the absence of microbial cues. Given that RESISTIN has been implicated in modulating inflammatory cytokine release, insulin sensitivity, lipid metabolism, and extracellular matrix remodeling, its elevated levels in SPF pigs may further contribute to enhanced metabolic activation and immune surveillance within endocrine microenvironments.

This study underscores the significant impact of microbiota absence on the cellular composition, gene expression, and intercellular communication in both adrenal and thyroid tissues. Our findings highlight a shift toward weakened immune activity and structural alterations in GF pigs, providing valuable insights into the functional adaptations of endocrine organs in the absence of a microbiota. These results emphasize the importance of using pigs as a more relevant model for studying the physiological consequences of microbiota absence in humans.

## CRediT authorship contribution statement

**Jingjing Tian:** Writing – review & editing, Writing – original draft, Resources, Data curation. **Teng Wang:** Visualization, Software, Resources, Investigation, Data curation. **Ting Zhou:** Writing – original draft, Visualization, Data curation. **Yu Pan:** Writing – original draft, Data curation. **Haitao Shang:** Resources, Validation. **Song Li:** Resources, Validation. **Shengjun Yang:** Validation, Resources, Data curation. **Li Li:** Supervision, Project administration, Resources. **Shuai Tan:** Writing – review & editing, Supervision, Resources, Project administration, Funding acquisition, Conceptualization. **Gaohui Zhu:** Writing – review & editing, Supervision, Resources, Project administration, Funding acquisition, Conceptualization.

## Ethics declaration

The study was approved by the Chongqing Academy of Animal Science Animal Ethics Committee (Approval number: IRB-20210167-R).

## Data availability

The raw scRNA-seq data have been uploaded to the National Genomics Data Center (NGDC) of China under accession OMIX012295.

## Funding

This work was supported by the 10.13039/501100012166National Key Research and Development Program (China) (No. 2021YFA0805904), the 10.13039/501100001809Natural Science Foundation of China (No. 82570627), Chongqing Municipal Mid-aged and Young Medical High-level Talents Project (China) (No. YXGD202579), and Postdoctoral Fellowship Program of CPSF (No. GZC20251464).

## Conflict of interests

The authors declare no competing interests.
