# Supplementary material for: Loss of microbial signals reprograms endocrine microenvironments and consistently reduces RESISTIN expression in the adrenal and thyroid cells of germ-free pigs
Source: Genes Dis. 2026 Feb 13;13(6):102078. doi: 10.1016/j.gendis.2026.102078 (PMC13319678; doi:10.1016/j.gendis.2026.102078)
Supplement: Multimedia component 1 [file mmc1.docx]

**Supplementary Figure legends**

**Figure S1** Quality control analysis of single-cell data from SPF and GF pigs. **(A)** Violin plots showing the distribution of five key quality control metrics for each sample before quality control, including nFeature_RNA, nCount_RNA, percent.mt, percent.rb, and percent.hb, in both SPF and GF pigs. **(B)** Principal Component Analysis (PCA) showing the distribution of cells from SPF and GF pigs, with different colors representing different samples. **(C)** Distribution of cells after batch effect correction using the Harmony algorithm, with color representing samples from SPF and GF pigs. **(D)** Elbow plot showing the variance explained by the principal components after Harmony batch effect correction for SPF and GF pig samples. **(E)** Visualization of cell contamination scores in UMAP reduced-space for SPF and GF pigs. Color intensity indicates the level of contamination, with darker colors representing higher potential for environmental RNA contamination. **(F)** Density distribution plot of cell contamination scores for SPF and GF pigs. The red dashed line (x = 0.15) represents the contamination threshold, with cells on the left being clean and cells on the right potentially contaminated. **(G)** UMAP visualization of integrated data from both SPF and GF pig samples, with colors distinguishing the samples. **(H)** Violin plots showing the distribution of five key quality control metrics for each sample after quality control in both SPF and GF pigs.

**Figure S2** Single-cell cell type annotation for SPF and GF pigs. **(A)** UMAP distribution of cells from SPF and GF pigs at a resolution of 0.3. **(B)** Expression levels of marker genes in each cell cluster from SPF and GF pigs, with color indicating the level of expression from red to blue, and dot size representing the proportion of cells expressing the corresponding gene within the cluster. **(C)** Expression of marker genes in annotated cell types from SPF and GF pigs.

**Method in Supplementary files**

**Single-Cell Data Processing and Analysis Methods**

In this study, single-cell transcriptomic data were analyzed using Seurat (version 5.2.1). First, after preliminary quality control of the gene expression matrix, mitochondrial genes (MT.genes, ATP6, ATP8, COX1, COX2, COX3, CYTB, ND2, ND3, ND4, ND4L, ND5, ND6), ribosomal genes (RB.genes, sourced from the article PMID: 35750885), and hemoglobin-related genes (HB.genes, HBB, HBE1, HBZ, HBM, HBQ1, EPB42, SPTA1, SPTB, SLC4A1, ANK1) were identified. The PercentageFeatureSet function in the Seurat package was then used to calculate the proportions of these three gene categories in each cell. Cells were filtered based on the following thresholds: 500–5000 detected genes, a total UMI count no greater than 15,000, mitochondrial gene proportion < 20%, and hemoglobin gene proportion < 0.5%. Additionally, decontX (version 1.6.0) was used to calculate and remove potential environmental RNA contamination, retaining only cells with a contamination ratio < 15%.

After quality control, the data were first normalized using NormalizeData, followed by the identification of highly variable genes using FindVariableFeatures (selection.method = "vst", nfeatures = 2000). To avoid bias, all MT.genes, RB.genes, and HB.genes were excluded from the highly variable gene list. The remaining highly variable genes were used for ScaleData and principal component analysis (PCA, npcs = 50), and batch effects were corrected across samples using Harmony (version 1.2.3). Based on the ElbowPlot results, the top 30 principal components were selected for subsequent dimensionality reduction and visualization using UMAP (RunUMAP) for 2D embedding.

Cell clustering was performed using Seurat: FindClusters, and cell type annotation was done at a resolution of 0.3 using well-known marker genes. The main marker genes included: T cells (CD2, CD3D, CD3E), B cells (CD37, MS4A1, CD79A), Monocytes (CD14, LYZ), Macrophages (CD68, C1QA, C1QC), Endothelial cells (PECAM1, VWF, LYVE1), Fibroblasts (COL1A1, MFAP4, MGP), Smooth muscle cells (ACTA2, CNN1, MYL9), Cortical cells (CYP21A2, STAR), Follicular epithelial cells (TG, TPO, TSHR), Chromaffin cells (CHGA, CHGB, TH), Neutrophils (SELL, S100A8, CSF3R), Steroidogenic-like cells (CYP11A1, FDX1, FDXR), and Cycling cells (PCNA, BIRC5). Differential gene analysis was performed using FindMarkers with thresholds set at min.pct = 0.1, and significant genes were selected with p_val_adj < 0.05 and |avg_log2FC| > 0.25. Differential genes were further subjected to functional enrichment analysis using clusterProfiler (version 4.16.4). Gene ID conversion was done using org.Ss.eg.db (version 3.20.2), and GSEA analysis was conducted using the enrich KEGG function.

**Cell–Cell Communication Analysis**

Cell**–**cell communication analysis of the single-cell transcriptomic data was performed using the CellChat (v2.2.0) R package. CellChat objects were constructed for the thyroid and adrenal glands of both GF and SPF pigs, using the normalized gene expression matrix and cell type annotation information. Since there is currently no available ligand**–**receptor database for pigs, we used the human-based CellChatDB.human's Secreted Signaling pathway to ensure cross-species comparability, filtering and smoothing the overexpressed genes and interactions. The communication probability was then calculated, low-quality interactions were filtered out, and aggregation and centrality analyses were performed at the signaling pathway level.

In the comparative analysis, the netVisual_diffInteraction function was used to display the differences in communication networks between GF and SPF pigs for both thyroid and adrenal glands based on interaction counts and interaction strength. The rankNet function was used to rank signaling pathways based on information flow or interaction counts. Finally, shared signaling pathways between the two groups were extracted, and the netAnalysis_signalingRole_heatmap function was used to visualize the dominant cell types for each signaling pathway.
