# Supplementary figures and images for: Loss of microbial signals reprograms endocrine microenvironments and consistently reduces RESISTIN expression in the adrenal and thyroid cells of germ-free pigs
Source: Genes Dis. 2026 Feb 13;13(6):102078. doi: 10.1016/j.gendis.2026.102078 (PMC13319678; doi:10.1016/j.gendis.2026.102078)

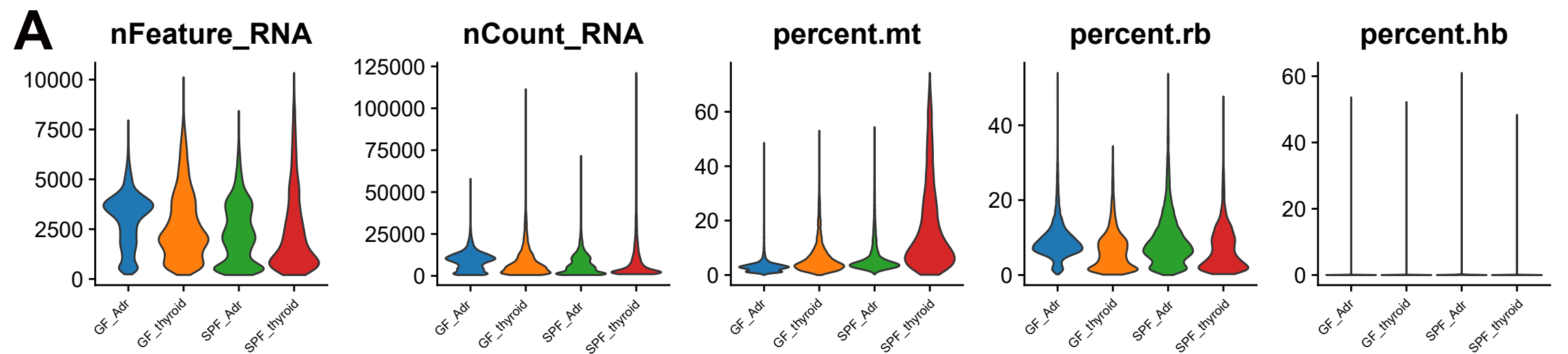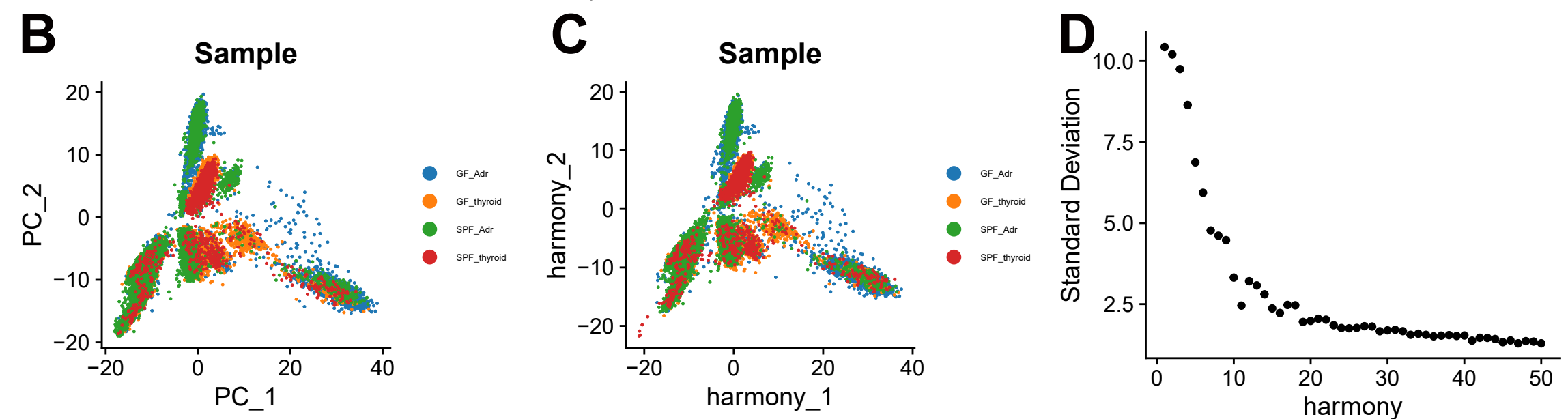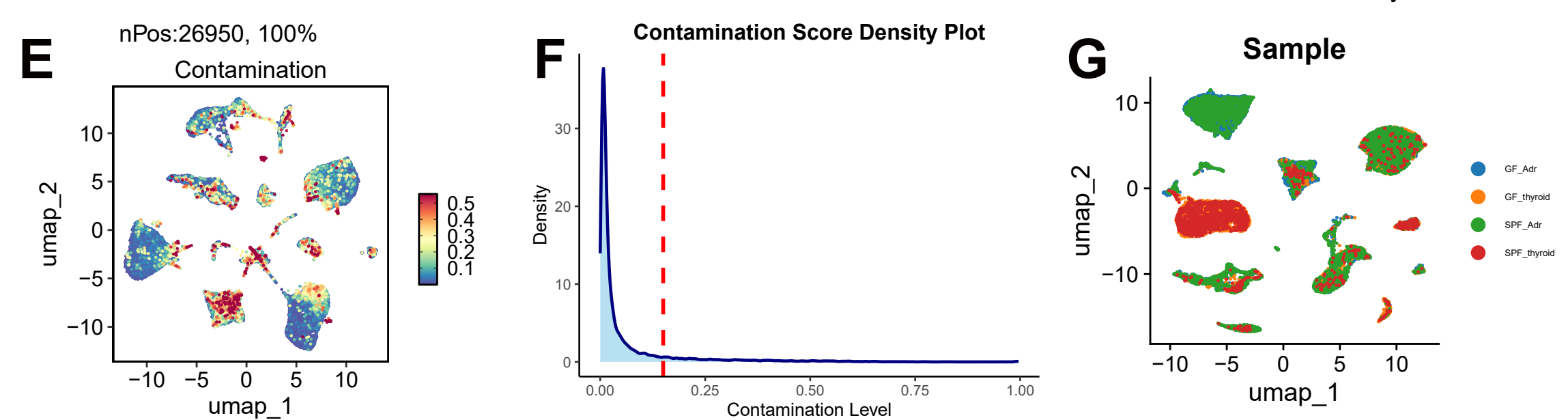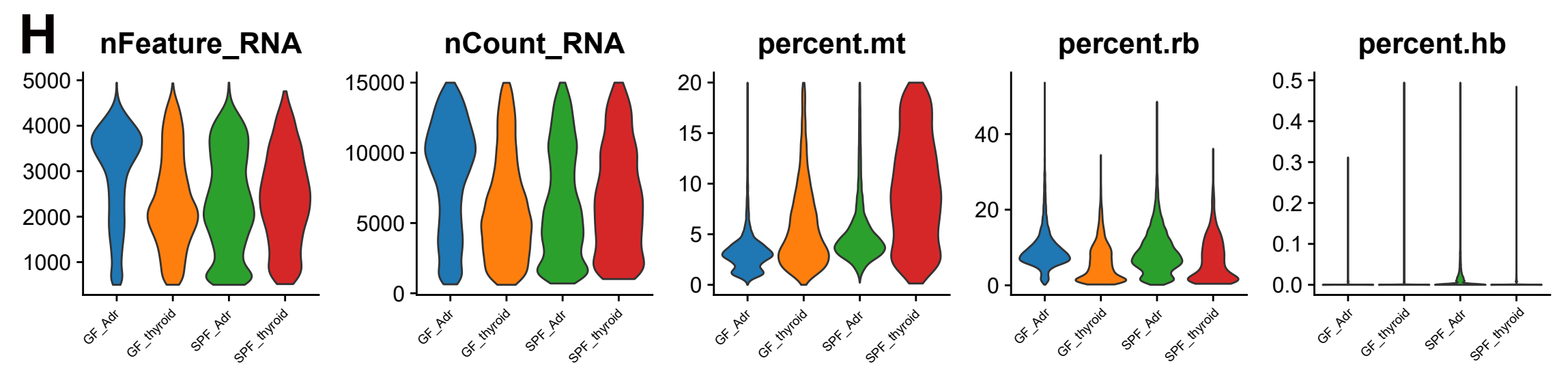

Supplement: Multimedia component 2 [file mmc2.pdf]

**A**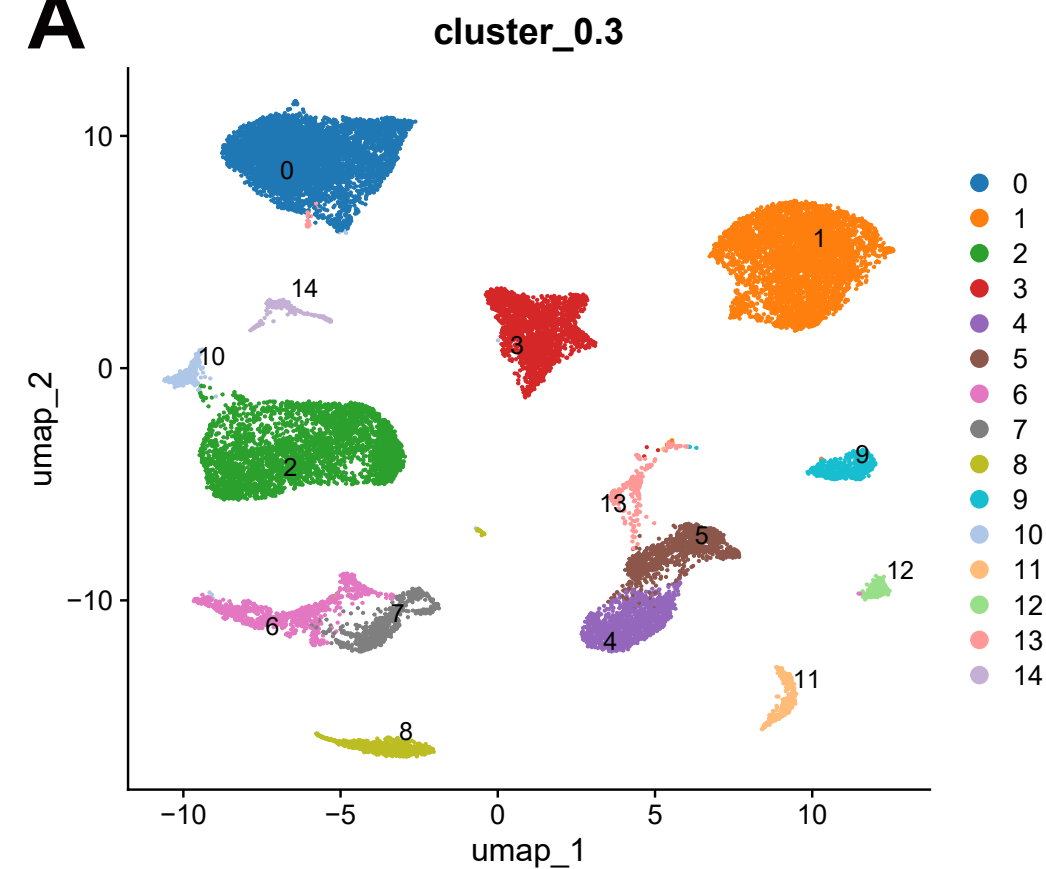**B**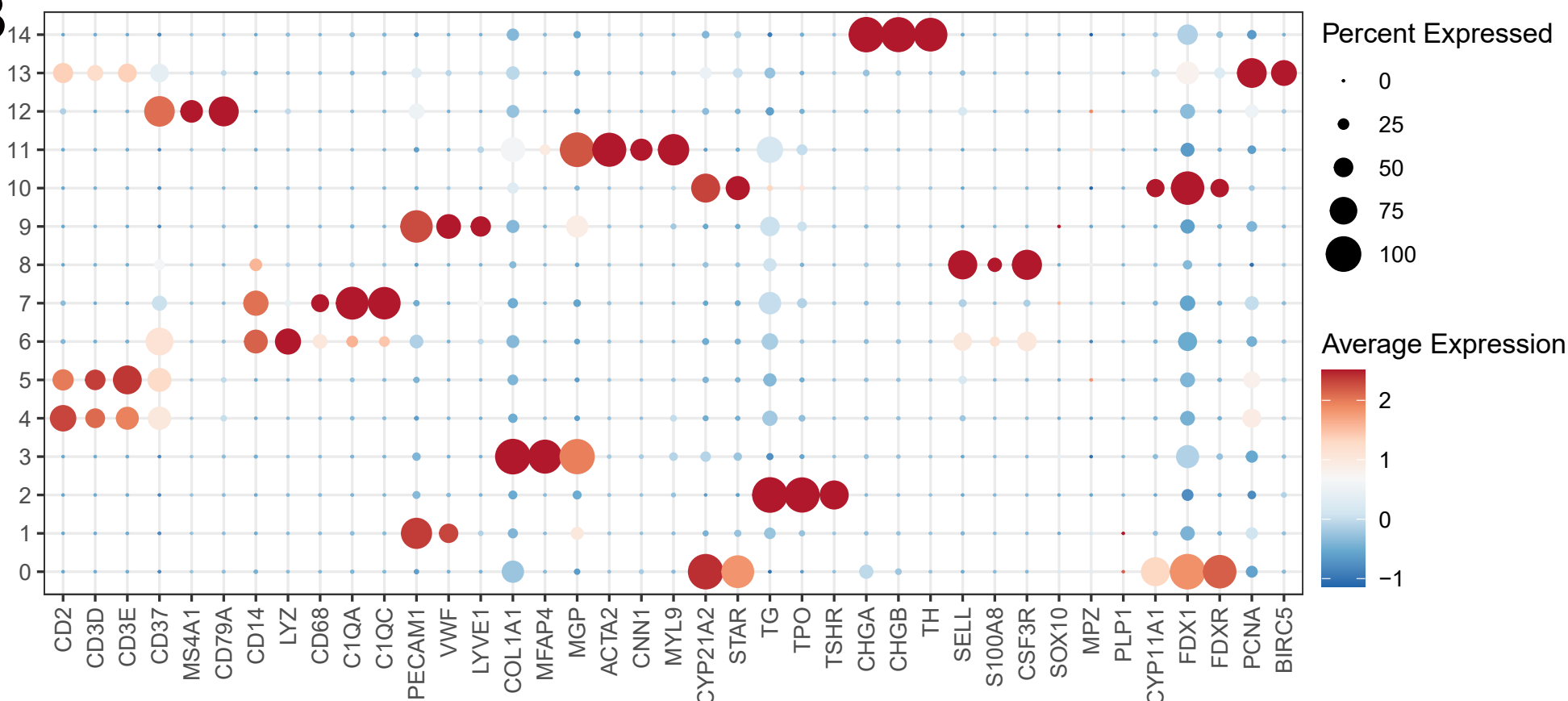**C**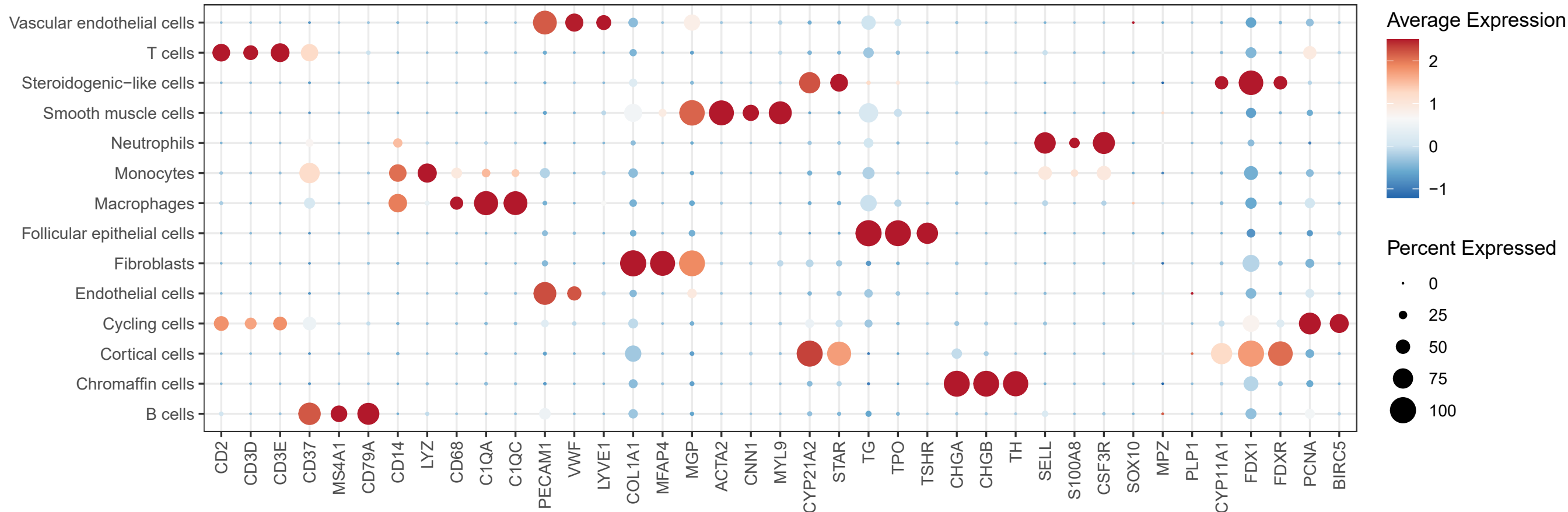

Supplement: Multimedia component 3 [file mmc3.pdf]
